# PET/CT imaging detects intestinal inflammation in a mouse model of doxorubicin-induced mucositis

**DOI:** 10.3389/fonc.2022.1061804

**Published:** 2022-12-15

**Authors:** Sina Dalby, Sofie Skallerup, Christina Baun, Lene Gaarsmand Christensen, Mathias Rathe, Mikael Palner, Steffen Husby, Jesper Bonnet Moeller

**Affiliations:** ^1^ Hans Christian Andersen Children’s Hospital, Odense University Hospital, Odense, Denmark; ^2^ Department of Clinical Research, University of Southern Denmark, Odense, Denmark; ^3^ Department of Cancer and Inflammation Research, Department of Molecular Medicine, University of Southern Denmark, Odense, Denmark; ^4^ Department of Nuclear Medicine, Odense University Hospital, Odense, Denmark; ^5^ Department of Pathology, Odense University Hospital, Odense, Denmark; ^6^ Danish Institute for Advanced Study, University of Southern Denmark, Odense, Denmark

**Keywords:** gastrointestinal mucositis, PET/CT, chemotherapy, doxorubicin, 2-[^18^F]FDG, mice

## Abstract

**Introduction:**

A severe side effect of cancer chemotherapy is the development of gastrointestinal mucositis, characterised by mucosal inflammation. We investigated if 2-deoxy-2-[^18^F] fluoro-D-glucose positron emission tomography combined with computed tomography (2-[^18^F]FDG-PET/CT) could visualise gastrointestinal mucositis in mice treated with the chemotherapeutic agent doxorubicin.

**Methods:**

In this study, gastrointestinal inflammation was longitudinally evaluated by 2-[^18^F]FDG-PET/CT scans before and 1, 3, 6, and 10 days after treatment with doxorubicin. Doxorubicin-treated mice were compared to saline-treated littermates using the abdominal standard uptake value of 2-[^18^F]FDG corrected for body weight (SUV_BW_).

**Results:**

Abdominal SUV_BW_ was significantly increased on day 1 (p < 0.0001), day 3 (p < 0.0001), and day 6 (p < 0.05) in the doxorubicin-treated group compared to controls. Abdominal SUV_BW_ returned to baseline levels on day 10. In the doxorubicin group, the largest weight loss was observed on day 3 (control *vs* doxorubicin, mean percent of baseline weight: (98.5 ± 3.2% *vs* 87.9 ± 4.6%, p < 0.0001). Moreover, in the doxorubicin-treated group, villus lengths were decreased by 23-28% on days 1 and 3 in the small intestine (p < 0.05), and jejunal levels of tumour necrosis factor and interleukin-1β were significantly increased on day 3 (p < 0.05).

**Discussion:**

Together, these findings indicate that sequential 2-[^18^F]FDG-PET/CT scans can objectively quantify and evaluate the development and resolution of intestinal inflammation over time in a mouse model of doxorubicin-induced mucositis.

## 1 Introduction

Chemotherapy-induced mucositis is an inflammatory process that affects the mucosal surfaces of the alimentary tract and leads to structural, functional, and immunological changes (1). It can be subdivided into oral mucositis and gastrointestinal mucositis. Previous research has focused on oral mucositis, probably due to the easier access to the oral cavity, but gastrointestinal mucositis has gained increasing attention due to recognition of the impact of antineoplastic therapy on the entire gastrointestinal tract (1–3).

Many patients experience some degree of mucositis during cancer treatment, which is often a substantial health issue for the individual patient (4, 5). Patients with mucositis experience fatigue, nausea, diarrhoea, abdominal pain, reduced oral intake, and weight loss (6). Bacterial translocation across the injured mucosa frequently leads to bacteraemia and sepsis (6). Chemotherapy regimens induce various degrees of mucositis depending on the drugs involved, the cancer diagnosis and stage, and patient-specific factors such as age and gene variants related to drug metabolism and cell repair pathways (7–9). The diagnosis of gastrointestinal mucositis currently depends on subjective measures, such as pain and diarrhoea, which are influenced by other clinical factors that can be difficult to asses in young children (2, 10). Objective measures specifically for gastrointestinal mucositis are lacking.

The currently accepted model for the development of mucositis was proposed by Sonis (11). This model describes five phases through which mucositis develops and dissolves in an interplay between the epithelial cells and the underlying tissue. Briefly, the mucositis model outlines phases 1) Initiation with DNA and non-DNA damage, 2) Primary damage response with activation of transcription factors, 3) Signal amplification with the production of pro-inflammatory cytokines, 4) Ulceration involving an influx of immune cells into the mucosa and breakdown of the epithelial barrier, and 5) Healing of the epithelium (11–13).

Several animal models have been developed for studying the pathophysiology, development, and possible treatments of gastrointestinal mucositis. Each model has its advantages and disadvantages related to the degree of translatability to human conditions, immunology, physiology, and microbiology, as well as the analytical techniques used and the research feasibility (14, 15). An animal model of gastrointestinal mucositis caused by doxorubicin has proven suitable for mucositis research (16, 17). Weight loss is often used as a marker of mucositis development (15), but it is unspecific and does not determine the location of mucositis in the gastrointestinal tract. Techniques that determine the location of mucositis in the gastrointestinal tract require the termination of the animals to obtain tissue for analyses, and reliable *in vivo* modalities for evaluating chemotherapy-induced gastrointestinal toxicity are lacking. Using a non-invasive molecular imaging modality would mean that each animal could be examined at several time points and thus serve as its own control. This would reduce inter-animal variability and the number of animals needed in a study. 2-deoxy-2-[^18^F] fluoro-D-glucose (2-[^18^F]FDG) positron emission tomography (PET) visualises inflammation on a molecular level in the entire gastrointestinal tract and identifies areas of inflammation, which can be challenging to assess. Visualising gastrointestinal inflammation in animal models has been shown in infection and aseptic inflammation. High dose radiation has been shown to markedly increase 2-[^18^F]FDG uptake in the small intestine correlating with blunting of the villi (18). Yamato and co-workers have shown that 2-[^18^F]FDG PET was able to visualise small bowel ulceration caused by non-steroidal anti-inflammatory drugs (NSAID) in rats (19). In mice with severe colonic clostridium difficile infection, the marked increase in 2-[^18^F]FDG uptake correlated with the clinical condition (20). To our knowledge, the characteristics and dynamics of chemotherapy-induced mucositis over time has not previously been studied with PET.

In this study, we investigated whether 2-[^18^F]FDG-PET/CT is a useful modality for visualising and semi-quantifying mucositis development in the intestines after single-injection doxorubicin therapy, which causes mild mucositis. Specifically, we investigated whether 2-[^18^F]FDG-PET/CT can identify an increased abdominal 2-[^18^F]FDG uptake in doxorubicin-treated mice compared to saline-treated controls and monitor this change over time. The presence of intestinal mucositis was evaluated by changes in weight, intestinal length, histomorphologically, and gene expression of inflammatory markers in the intestinal tissue. The data obtained are likely to be directly applicable to humans with mucositis and may help to establish new diagnostic and therapeutic avenues.

## 2 Materials and methods

### 2.1 Setting and ethics

Experiments were conducted at the Preclinical Imaging Core Facility, University of Southern Denmark, Odense, Denmark. All animals had a 2-week acclimatisation period in a dedicated animal housing facility at the Biomedical Laboratory of the university. Mice were housed 3-4 together in disposable cages with ALPHA-dri Dust-free bedding and had access to enrichment and food ad libitum, except during the fasting period prior to each scanning. The mice were housed with a 12 h light and 12 h dark cycle at 20–25°C, and water was accessible at all times. The mice were weighed daily, either in the morning or just before each scanning session and were inspected daily by experienced animal caretakers for any signs of pain or other symptoms. The Danish Animal Experiments Inspectorate approved the study (licence number: 2017-15-0201-01385), which was performed in compliance with the PREPARE guideline (21) and reported according to the ARRIVE 2.0 guideline (22). The study protocol for the scanned mice was registered at preclinicaltrials.eu (registry ID: PCTE0000188).

### 2.2 Mice and study design

**Figure 1:**
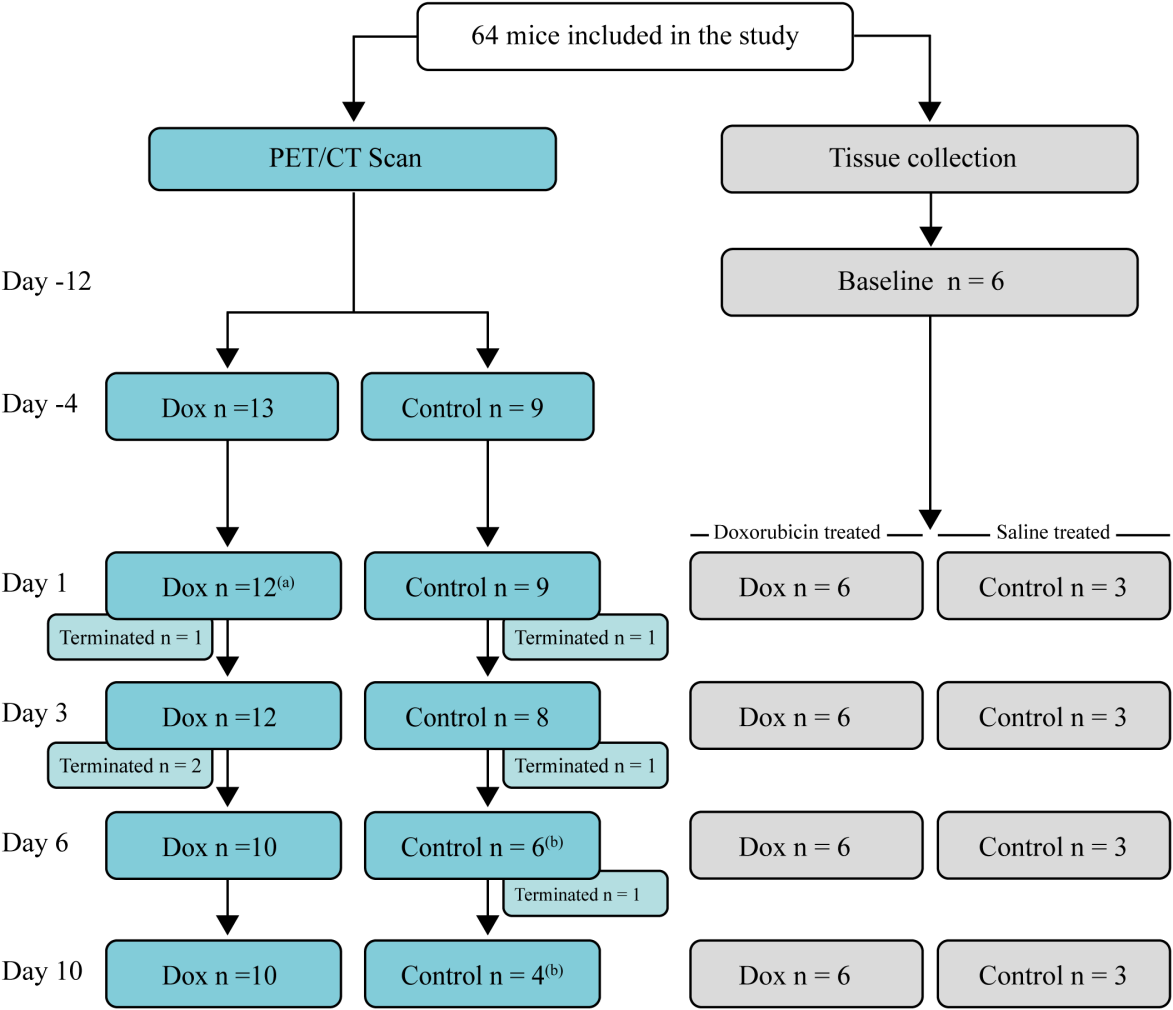
Study flowchart and timeline. Days of data collection (either 2-[^18^F]FDG PET/CT scans or collection of specimens for analysis) relative to the day of doxorubicin or saline injection (day 0). Left: Mice were scanned sequentially, and after each scan, a few mice (n = 1-2) were terminated for tissue collection. Right: Mice used for tissue collection. The number of mice, terminated at each time point is indicated. Mice in the tissue collection group underwent the same procedures as the mice that were scanned, except for the scan. (^a^) One mouse was erroneously not scanned. (^b^) A mouse died in relation to the 2-[^18^F]FDG injection.

Female C57BL/6n age-matched (9-12 weeks, 18-24 g) littermates were purchased from Taconic Biosciences (Rensselaer, New York USA). Mice were allocated to either a scan group subjected to sequential 2-[^18^F]FDG-PET/CT scans or a parallel group that underwent the same procedures except for the scan. The mice were randomised to either saline or doxorubicin treatment. The animals in each cage were allocated to the same experimental group, as mice from different groups could not be housed together. Confounders such as cage placement were not controlled. Sixty-four mice were included in the study. Six mice were randomly chosen to obtain tissues for baseline analyses 12 days before doxorubicin or saline injection. **Figure 1** describes the study timeline.

### 2.3 Doxorubicin-induced mucositis

Mucositis was induced by an intraperitoneal injection of doxorubicin (Accord Healthcare, Devon UK), diluted 1:1 with isotonic saline at a dose of 15 mg/kg. Mice in the control group received an equal volume of isotonic saline. The humane endpoint was set *a priori* at a 20% weight loss. If mice treated with doxorubicin lost less than 5% body weight, they were excluded due to assumed injection error as previously described (23, 24). Weight was measured as the percent change versus baseline and presented as mean ± standard deviation.

### 2.4 PET/CT imaging with 2-[^18^F]FDG

Two days before each scan, the mice were provided CT contrast (Iomeron, 23 mg iodine/ml) in artificially sweetened (0.004 g/mL Sweet’N Low, Bernard Food Industries, Illinois USA) drinking water. The mice were fasted a minimum of four h before each scan to ensure low blood sugar. The mice were anaesthetised with a mixture of 1.5-2% isoflurane and 100% oxygen before injection of 0.2 ml bolus 2-[^18^F]FDG into the tail vein. The injected dose was 49 MBq (standard deviation 2.6). Blood glucose was measured immediately before administration of 2-[^18^F]FDG. The mice were kept under light anaesthesia for 30 min post-injection of 2-[^18^F]FDG to prevent muscle uptake and were then returned to their cage for 60 min for the opportunity to empty their bladder. The mice were kept warm during all handling procedures.

PET/CT imaging was performed on a Siemens INVEON multimodality preclinical scanner in a docked mode (Siemens, Knoxville, Tennessee, USA). During each imaging session, the mice were re-anaesthetised with a mixture of 1.5-2% isoflurane and 100% oxygen 90 min after injection of 2-[^18^F]FDG and placed feet first in a prone position on a dedicated animal bed (38 mm). The respiration and temperature of the animal were monitored during the imaging session using the BioVet system (M2M Imaging, Cleveland, Ohio, US).

The imaging protocol included a two-bed CT for anatomic orientation performed with full rotation, 360-degree projections, and exposure of 80 kV and 500 µA. A static PET scan immediately followed the CT scan with a duration of 30 min. CT and PET images were co-registered using a transformation. Reconstruction of PET data was performed using an OSEM3D/SP-MAP algorithm (2 x OSEM iterations and 18 x MAP iterations) with scatter correction and a matrix size 128x128, resulting in a final target resolution of 1.5 mm.

### 2.5 Image analysis

Decay-corrected 2-[^18^F]FDG-PET/CT scans were analysed in PMOD version 4.2 (PMOD Software, RRID : SCR_016547, PMOD Technologies LLC, Zürich, Switzerland) without attenuation correction. One investigator assessed all scans. On PET images, a spherical volume of interest (VOI) was placed covering the abdominal region. The physiologically high 2-[^18^F]FDG uptake in kidneys and bladder was identified based on anatomical location and appearance and was manually subtracted from the VOI. Areas of the VOI without uptake were also manually removed. The resulting VOI was transferred to the CT images to ascertain that no skeletal structures were included and that all gastrointestinal contrast in the small intestine was included. VOI delineation was evaluated in the sagittal, coronal, and transverse planes. Mean standard uptake values corrected for body weight (SUV_BW_) from the entire VOI were used in the analysis (an example of VOI delineation is presented in **Supplementary Figure S1**).

### 2.6 Tissue collection

For the collection of tissue specimens, mice were terminated under deep anaesthesia. The intestines were excised at the pyloric sphincter and rectum and were then cleaned and photographed. Images were analysed with ImageJ (U.S. NIH, Bethesda, Maryland USA) to measure the length of the small intestine from the pyloric sphincter to the ileocaecal junction and the large intestine from the ileocecal junction to the rectum. The stomach and spleen were weighed, and the stomach was opened, flushed with phosphate-buffered saline (PBS), and gently dried before weighing.

### 2.7 Histomorphology

Intestinal segments corresponding to the duodenum (3-4.5 cm from the pyloric sphincter), jejunum (from the middle part of the small intestine), and ileum (4.5-6 last cm before the caecum) were excised, cleaned, and fixed in 4% formaldehyde at room temperature for 24 h. After fixation, each piece was divided into three segments and stored in Dulbecco’s PBS at 4°C before paraffin embedding and sectioning. Samples were stained with Haematoxylin and Eosin (H&E) and analysed using NDP.view2 Image viewing software (Hamamatsu Corporation, Bridgewater, New Jersey USA). In the small intestinal tissue specimens, villi were measured from the crypt-villus junction to the top of the villi with a single column of cells along the border. Adjacent crypts were measured from the crypt-villus junction to the bottom of the crypt. 5-10 villus-crypt pairs were measured in each segment, and the average heights and depths were calculated. Finally, the villus-crypt ratios and the average for each segment were calculated. Grading of the mucosal damage for the jejunal tissue specimens was performed by an experienced pathologist as previously described (25): Grade 0: Normal mucosal villi. Grade 1: Development of the subepithelial Gruenhagen’s space. Grade 2: Extension of the subepithelial space with a moderate lifting of the epithelium. Grade 3: Massive epithelial lifting down the sides of the villi. Grade 4: Denuded villi with lamina propria and dilated capillaries exposed. Grade 5: Digestion and disintegration of the lamina propria.

### 2.8 Real-time quantitative polymerase chain reaction

Intestinal tissue samples were gently flushed with ice-cold PBS before being transferred to tubes containing 1 mL of TRIzol Reagent (Ambion Life Technologies, Invitrogen, Carlsbad, California, USA) and stored immediately after the procedure at –80°C. Tissues were homogenised using a Precellys^®^ 24 homogeniser (Bertin Instruments, Montigny-le-Bretonneux, France), followed by RNA purification according to the manufacturer’s instructions. Nucleic acid yields were measured on a NanoDrop™ One Spectrophotometer (Thermo Fisher Scientific, Waltham, Massachusetts, USA). The A260/A280 ratio was > 1.9 for all RNA samples. 2 µg RNA was used for cDNA synthesis using the High-Capacity cDNA Reverse Transcriptase kit according to the manufacturer’s recommendations (Applied Biosystems, Waltham, Massachusetts, USA). Quantification of gene expression was performed on a qTOWER3 instrument with qPCRsoft software (both Analytik Jena, Jena, Thuringia, Germany) using the following TagMan gene expression assays: mouse *Tnf* (Mm00443258_m1), *Il-1β* (Mm00434228_m1) and *Hprt1* (Mm00446968_m1) obtained from Applied Biosystems, (Waltham, Massachusetts, USA). Gene expression was normalised as the n-fold difference to *Hprt1* according to the cycling threshold.

### 2.9 Blinding

No blinding was employed during mouse allocation and conduction of the experiment. Outcome assessment of scan data, histomorphology, and inflammatory markers was blinded.

### 2.10 Statistical methods

The normality of the data distribution was evaluated visually and with the Shapiro-Wilk test. Normally distributed data are presented as mean ± standard deviation (sd), and non-normally distributed data are presented as median and interquartile range (IQR). Histomorphology and bowel lengths were compared using a two-way ANOVA followed by Holm-Šídák’s multiple comparisons test. Gene expression of *Tnf and Il-1β* were analysed with multiple Mann-Whitney tests, where the multiplicity adjusted p-value was reported. Repeated measures of body weight and abdominal SUV_BW_ were analysed using a mixed model with Geisser-Greenhouse correction (as sphericity is likely to be violated in this longitudinal study), followed by Holm-Šídák’s multiple comparisons test. Statistical significance was defined as p < 0.05. Statistical analyses were performed using GraphPad Prism version 9.3.1 (GraphPad Software, San Diego, California USA).

## 3 Results

Results were pooled from two independent experiments with similar results. The numbers of mice included in the analyses are presented in **Figure 1**. One mouse in the doxorubicin group was excluded due to a weight loss of less than 5%. Two mice from the control group died immediately after 2-[^18^F]FDG injection on days 6 and 10, respectively, most likely due to air bolus injection. One mouse was not scanned on day 1 due to a replacement error. At the end of the experiment on day 10, all remaining mice were terminated, and specimens were collected for analysis.

### 3.1 Treatment with doxorubicin resulted in significant weight loss

**Figure 2:**
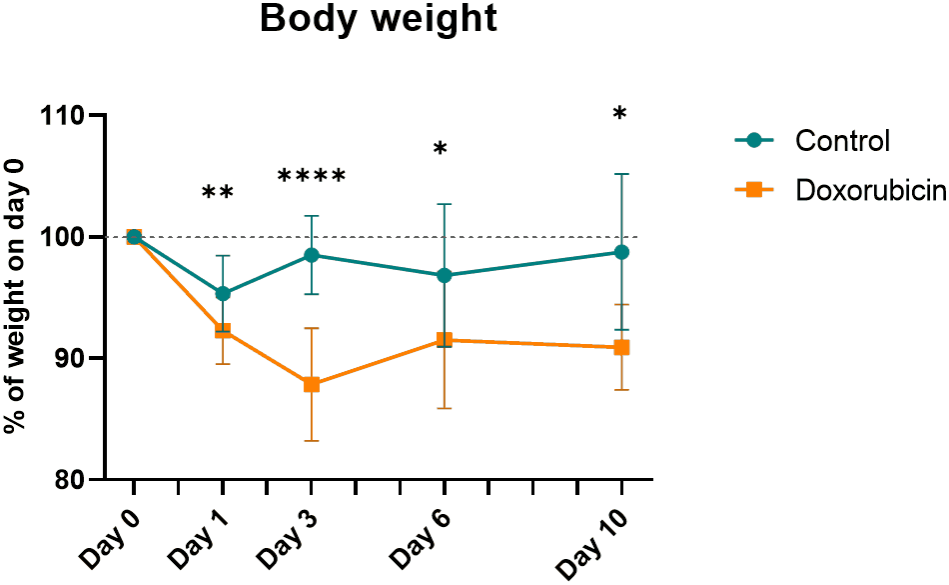
Body weight normalised to weight at day 0. For controls: Day 0 and 1, n = 21; day 3, n = 17; day 6, n = 13; day 10, n = 8. For doxorubicin: day 0 and 1, n = 37; day 3, n = 30; day 6, n = 22; day 10, n = 16. Data presented as means ± sd. *p ≤ 0.05, **p ≤ 0.01, ****p ≤ 0.0001.

Mice in the doxorubicin-treated group lost significantly more weight than the control group from day 1 until day 10 (control *vs*. doxorubicin, percent of baseline weight; day 1 (95.3 ± 3.1% *vs*. 92.3 ± 2.8%, p = 0.0019); day 3 (98.5 ± 3.2% *vs* 87.9 ± 4.6%, p < 0.0001); day 6 (96.8 ± 5.9% *vs*. 91.5 ± 5.6%, p = 0.0202) and day 10 (98.8 ± 6.4% *vs*. 90.9 ± 3.5%, p = 0.0202)) (**Figure 2**).

### 3.2 2-[^18^F]FDG-PET detected intestinal mucositis in doxorubicin-treated mice

**Figure 3:**
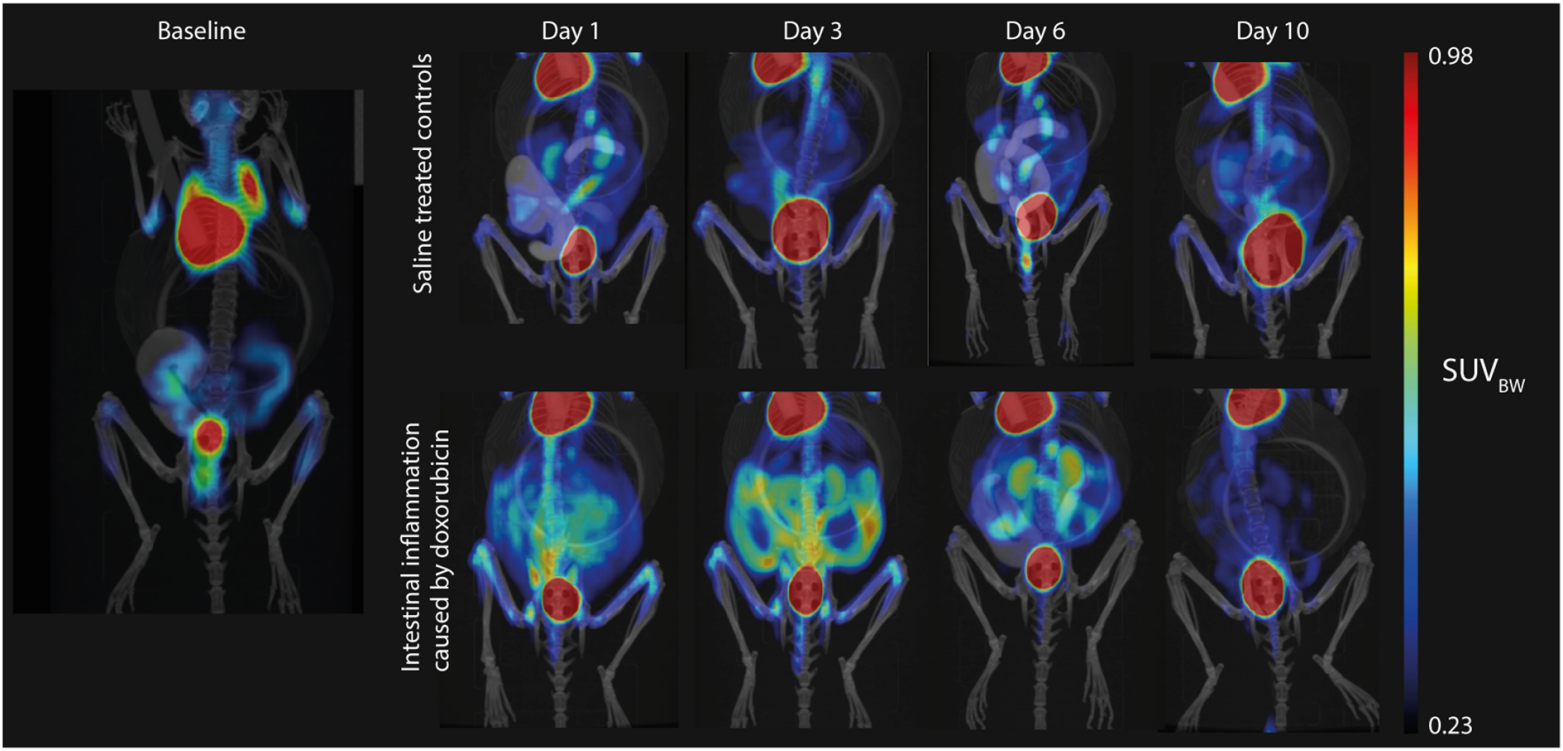
Representative examples of the 2-[^18^F]FDG uptake on the PET/CT scans. Uptake is very high in the heart and bladder. Left: Baseline hybrid PET/CT scan with low uptake in the intestines due to variable intestinal muscle activity. Upper panel: Sequential images from a mouse in the control group with consistently low intestinal uptake, which was comparable to baseline levels. Lower panel: Sequential images from a mouse in the doxorubicin-treated group with highly increased intestinal uptake on days 1 to 6.

**Figure 4:**
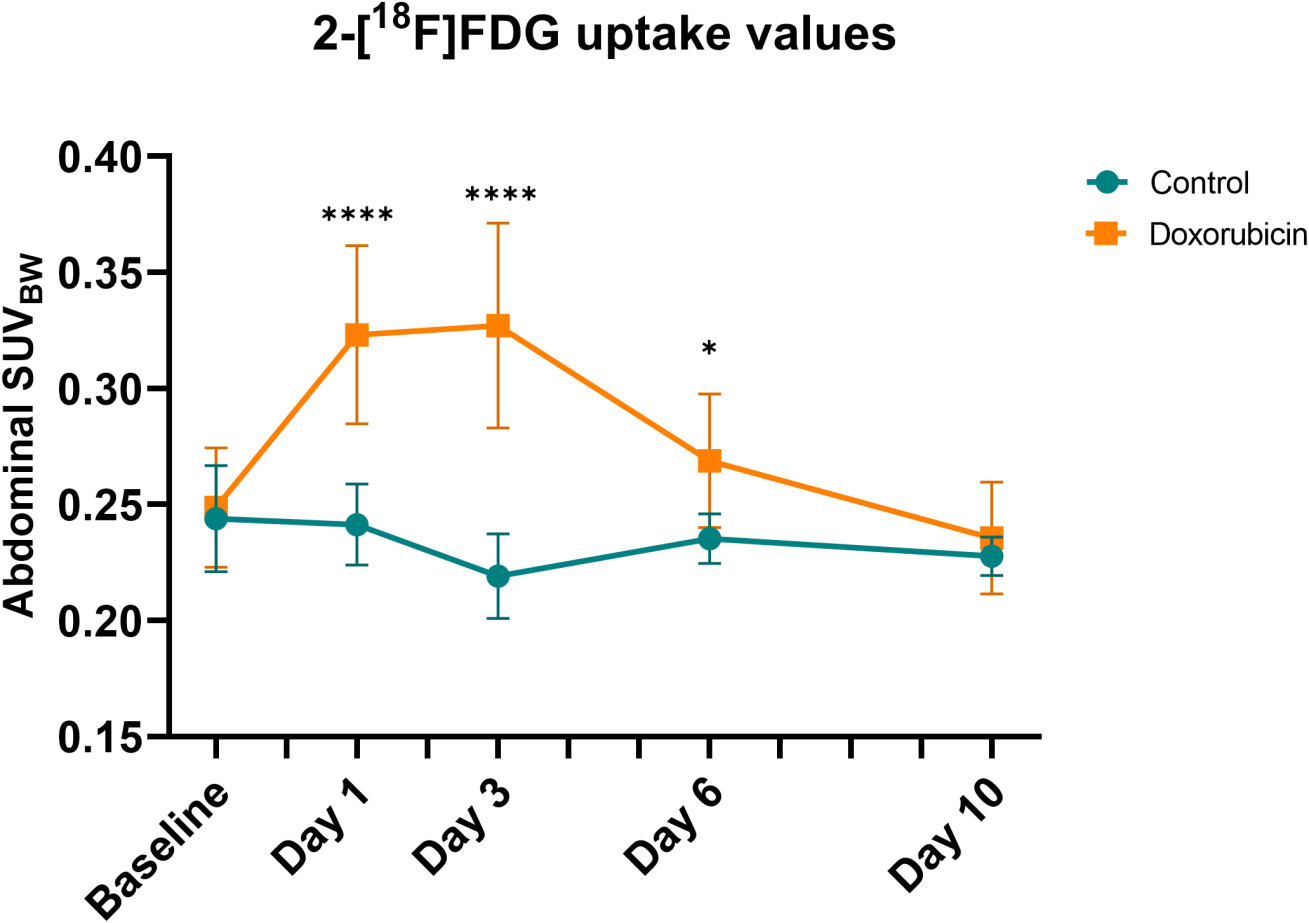
Abdominal standard uptake value corrected for body weight (SUV_BW_). In the doxorubicin-treated group, abdominal SUV_BW_ was increased on days 1 and 3 but returned to baseline levels by day 10. For controls: baseline and day 1, n = 9; day 3, n = 8; day 6, n = 6; and day 10, n = 4. For doxorubicin: baseline, n = 13; day 1 and 3, n = 12; day 6 and day 10, n = 10. Data are presented as means ± sd. *p ≤ 0.05, ****p ≤ 0.0001.

Representative images of mice from the control and doxorubicin groups are shown in **Figure 3**. Abdominal SUV_BW_ was significantly increased in the doxorubicin-treated group compared to the control group on days 1 through 6 (control *vs*. doxorubicin, day 1: 0.24 ± 0.02 *vs*. 0.32 ± 0.04, p < 0.0001; day 3: 0.22 ± 0.02 *vs* 0.33 ± 0.04, p < 0.0001; day 6: 0.24 ± 0.01 *vs* 0.27 ± 0.03, p < 0.05) and returned to baseline levels on day 10 (control *vs*. doxorubicin 0.23± 0.01 *vs*. 0.24± 0.02, p = 0.6) (**Figure 4**). The total uptake was calculated as average SUV_BW_ × VOI size. VOI size is depicted in **Supplementary Figure S5** and the total uptake is depicted in **Supplementary Figure S6**. The calculated total uptake was significantly greater in the doxorubicin group compared to the saline group on days 1-10 (control *vs*. doxorubicin, day 1: 1.023 ± 0.14 *vs*. 1.815 ± 0.32, p < 0.0001; day 3: 1.022 ± 0.14 *vs* 1.561 ± 0.16, p < 0.0001; day 6: 0.950 ± 0.12 *vs* 1.379 ± 0.20, p = 0.0002; day 10: 1.055 ± 0.14 *vs*. 1.320 ± 0.23, p = 0.0498). Blood glucose levels are presented in **Supplementary figure S7**.

### 3.3 Histomorphological changes in the intestinal mucosa were identified on days 1 and 3

**Figure 5:**
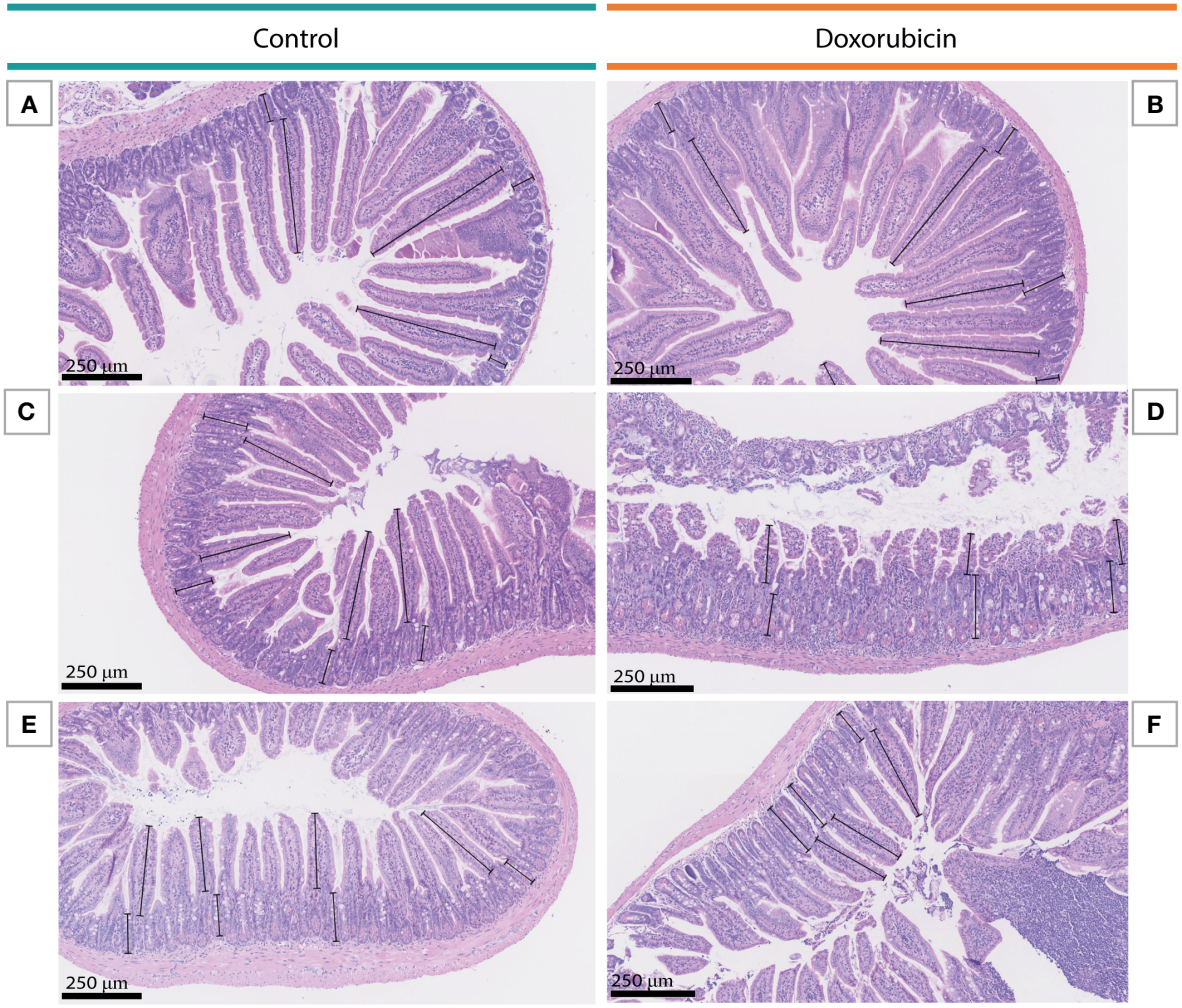
Examples of villus and crypt measurements in the small intestine on day 3. **(A, B)** Duodenal tissue from a mouse in the control group and a mouse in the doxorubicin group, respectively. **(C, D)** Jejunal tissue from a mouse in the control group and a mouse in the doxorubicin group, respectively. **(E, F)** Ileal tissue from a mouse in the control group and a mouse in the doxorubicin group, respectively.

**Figure 6:**
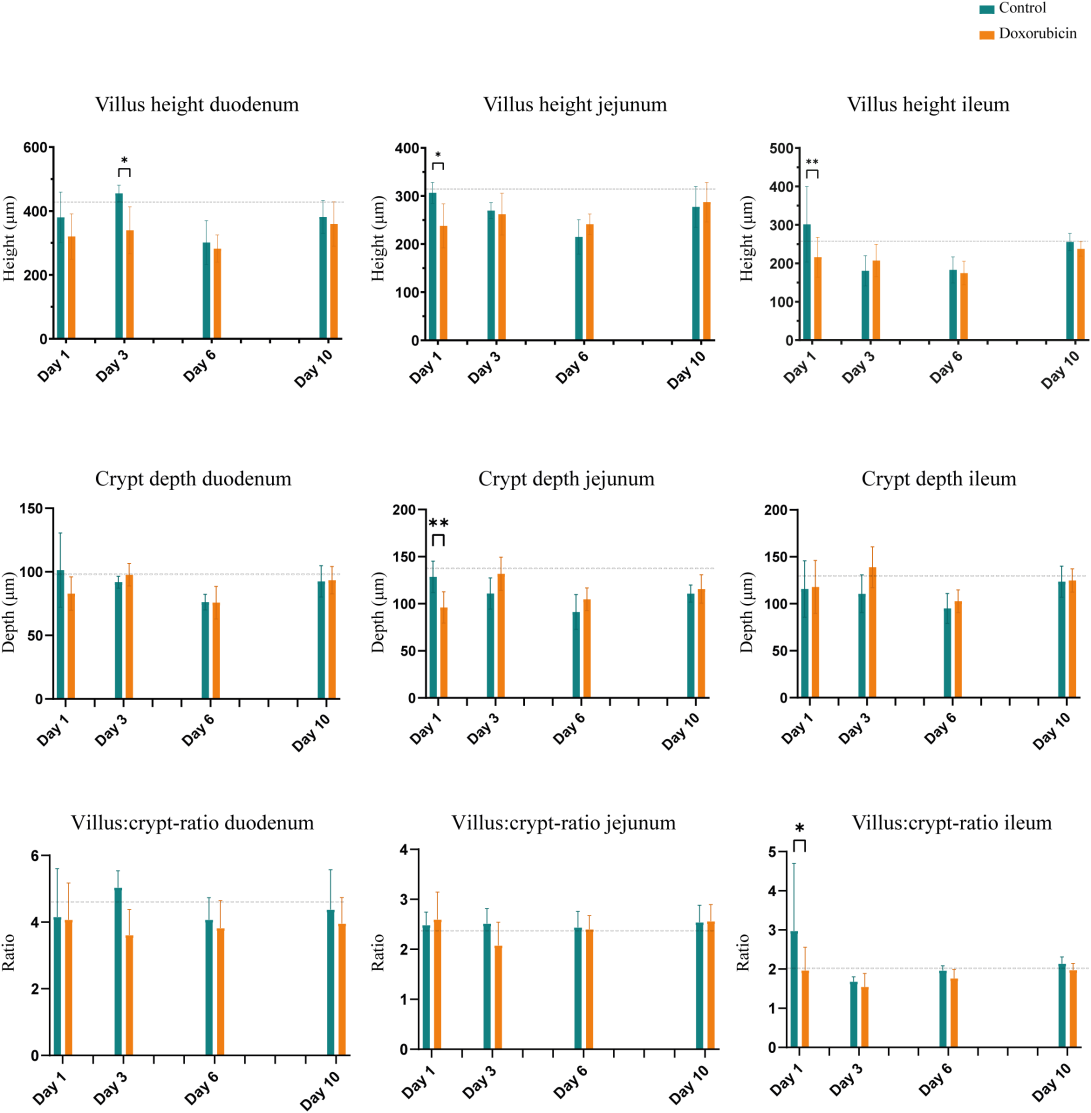
Histomorphological changes in the small intestine: Villus height, crypt depth, and villus:crypt-ratio. Baseline n = 6. For controls: day 1, n = 4; day 3, n = 4: day 6, n = 4; day 10, n = 7. For doxorubicin: day 1, n = 7; day 3, n = 8; day 6, n = 6; and day 10, n = 16. Data presented as mean ± sd. The dotted line represents the mean of the baseline values. *p < 0.05, **p < 0.01.

Doxorubicin treatment induced significant histomorphological changes on days 1 and 3 in the intestinal mucosa. **Figure 5** depicts examples of H&E-stained small bowel tissues with villus and crypt measurements. In the doxorubicin-treated group on day 1, villi were 22.5% shorter in the jejunum (control 307 ± 22 µm *vs* doxorubicin 238 ± 46 µm, p = 0.0244) and 28.2% shorter in the ileum (control 301 ± 98 µm *vs* doxorubicin 216 ± 52 µm, p = 0.006) compared to the control group. On day 3, there was a difference of 25.3% between the two groups in the duodenum (control 455 ± 25 µm *vs* doxorubicin 340 ± 73 µm, p = 0.0205). Crypts of the jejunum on day 1 were atrophic after treatment (control 129 ± 17 µm *vs* doxorubicin 96 ± 17 µm, p = 0.0056). On day 1, there was also a difference in the villus:crypt ratio of the ileum of 33.9% (control 2.98 ± 1.7 *vs* doxorubicin 1.97 ± 0.6, p = 0.012). Villus heights, crypt depths and villus:crypt ratios of the small intestine are presented in **Figure 6**. The histological grading system did not detect any significant differences between the two groups in the jejunal tissue samples. Treatment did not affect colonic crypts (data presented in **Supplementary Figure S2**). There were no significant differences between the groups in colon length, small intestinal length, spleen weight, or stomach weight on either day. Data are presented in **Supplementary Figures S3**, **S4**.

### 3.4 Doxorubicin induced a significant increase in expression of inflammatory markers on day 3

**Figure 7:**
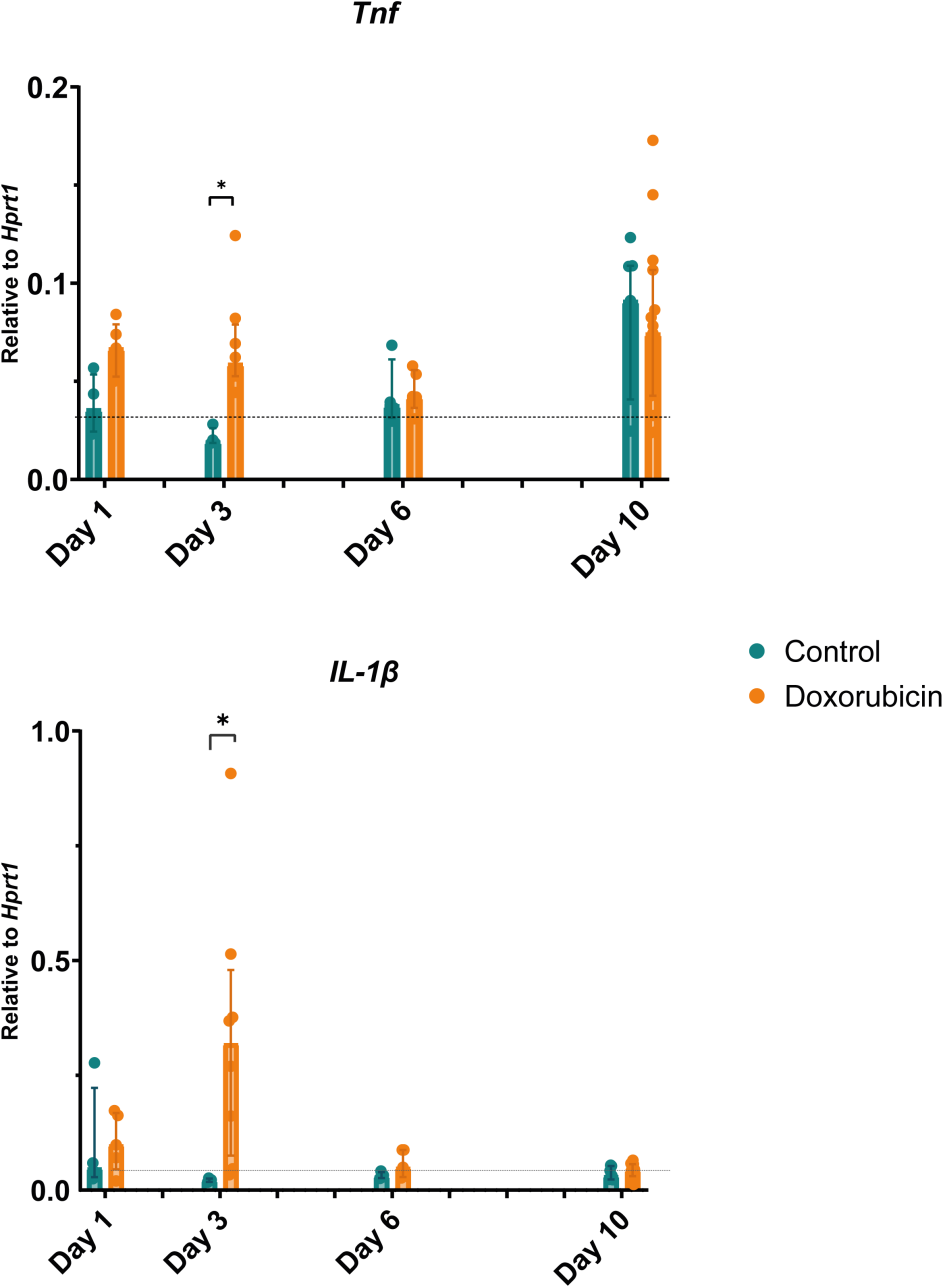
Changes in gene expression of inflammatory markers: tumour necrosis factor (*Tnf*) and interleukin (*Il*)-*1β*. On day 3, mice in the doxorubicin-treated group had elevated levels of *Tnf* and *Il-1β* compared to controls. Baseline, n = 6. For controls: day 1, n = 4; day 3, n = 4; day 6, n = 4: day 10, n = 7. For doxorubicin: day 1, n = 5; day 3, n = 5; day 6, n = 4; and day 10, n = 15. Data presented as scatter and bars representing the median ± interquartile range. The dotted line represents the mean of the baseline values. *p < 0.05.

The relative levels of both *Tnf* and *Il-1β* were increased in the doxorubicin-treated group on day 3 compared to the control group (**Figure 7**). *Tnf* levels were three-fold higher in the doxorubicin-treated group than in the control group (doxorubicin: 0.075 (IQR 0.063 to 0.112); controls: 0.025 (IQR 0.021 to 0.031), p = 0.016). The *Tnf* levels tended to be higher in the doxorubicin-treated group than in the control group on day 1, but this was not statistically significant (median 0.068 (IQR 0.053 to 0.086) *vs* 0.044 (IQR 0.029 to 0.063) p = 0.44). In the doxorubicin-treated group, *Il-1β* levels were highest on day 3 (0.319 (IQR 0.075 to 0.480) and were significantly higher than in the control group (0.022 (IQR 0.018 to 0.025) p = 0.016).

## 4 Discussion

This study suggested a time-dependent effect of chemotherapy-induced mucositis that was semi-quantifiable by 2-[^18^F]FDG PET/CT in the same individual assessed sequentially. Intestinal inflammation due to mucositis (measured by the abdominal SUV_BW_) was significantly increased on days 1, 3, and 6 and returned to baseline values on day 10. Weight loss was evident in the doxorubicin-treated group from day 1 through day 10 after doxorubicin treatment, and there was villus shortening and crypt atrophy on days 1 and 3 in the small intestine. Gene expression of the inflammatory markers *Tnf* and *Il-1β* was elevated on day 3.

The mice exhibited an expected weight loss, with mild histomorphological signs of mucositis in the small bowel with villus shortening and crypt atrophy. The cytokines *Il-1β* and *Tnf* have previously been implicated in the initiation and maintenance of mucositis *via* NF-kB (7, 26, 27). Previously increased mucosal levels of *Tnf* and *Il-1β* have been found in this model (17), although the results are conflicting (28, 29) The increased intestinal expression of *Tnf* and *Il-1β* on day 3 in the current model corroborated the presence of NF-kB mediated mucosal inflammation. Previously, increased mucosal levels of *Tnf* and *Il-1β* have been found in this model (17), although the results are conflicting (28, 29). Conflicting evidence regarding tissue cytokines may be a result of chemotherapy-depleted intestinal cells producing *Il-1β* and *Tnf* at certain time points. As pointed out earlier, the interacting effects and time-dependent sequence of events among cytokines, chemokines, and tight junction proteins and others factors may explain that these mediators show a consistent response to cytotoxic regimens across different studies (14, 30). The levels of *Tnf* exhibited a substantial variance on day 10, which we hypothesise to be caused by the repeated anaesthesia and scanning procedures performed on the mice surviving for 10 days. Despite relatively mild signs of mucositis, mucositis was indeed present and detected by PET/CT. This murine mucositis model can be used to examine the ulcerative and healing phases of mucositis. In the ulcerative phase, immune cells such as macrophages, adaptive immune cells, and lymphoid cells are present in the mucosa (31), creating the potential for identification using 2-[^18^F]FDG-PET (32). A preclinical model enables the testing of specific hypotheses under controlled conditions and with a minimal inter-individual variation. Still, there are challenges to translatability due to biological and pathobiological differences between humans and animals and the diversity of chemotherapy treatments and supportive cancer therapies (14).

Several groups have studied animal models using 2-[^18^F]FDG-PET/CT. A well-established and reproducible murine model of colitis is the dextran sodium sulphate (DSS)-model, in which mice are given DSS in their drinking water, leading to the development of acute or chronic inflammation depending on the experimental setup (33). Hindrykx and co-workers found a strong correlation between colonic 2-[^18^F]FDG uptake on PET scans and histological score as well as activated neutrophils, indicated by myeloperoxidase (MPO) levels, after 7 days of 4% DSS administration. They observed maximum inflammation on day 7 and recovery towards baseline levels on day 14 and found a significant reduction in PET signal after treatment with dimethyloxalylglycine (a hydroxylase inhibitor shown to protect against colitis) compared to placebo on day 7 (34). Another study using the DSS mouse model also found a significant correlation between 2-[^18^F]FDG uptake and histological damage, most pronounced in the middle of the colon (35). In these studies, the weight loss was considerably more prominent than in our study, suggesting a more severe disease state. The DSS model is well known to cause significant inflammation by a direct toxic effect on the epithelium localised to the colon (36), which may make it easier to quantify using PET/CT as the colon is more fixed in position in the abdominal cavity than the small intestine. In comparison, our mucositis models caused a more physiological, mild small intestinal inflammation through complex epithelial and non-epithelial interactions. Even so, we were still able to detect and semi-quantify the inflammatory response in this mucositis model. Another model of colitis in the rat is topical administration of 2,4,6-trinitrobenzene sulfonic acid (TNBS) in 50% ethanol, which induces transmural inflammation. A longitudinal 2-[^18^F]FDG PET/CT study showed that 2-[^18^F]FDG uptake increased as colitis developed after TNBS application and decreased as recovery ensued on day 15 (37). These results are in line with ours, however, SUV_max_ measurements used in this study are prone to be affected by noise (38) and did not incorporate the extent of the disease, as was achieved in our study. A characteristic of these models of gastrointestinal inflammation is that they are restricted to the colon as opposed to our mucositis model which primarily affects the small intestine. In rats, Yamato et al. showed that the formation of ulcers in the small intestine caused by NSAID can be detected with PET/CT. They found that mean of SUV_max_ was increased threefold on day 1 compared to pre-treatment levels and returned to baseline levels by day seven (19). The approach to quantify small intestinal ulcers, however, was not suitable to the diffuse inflammation found in chemotherapy-induced mucositis. Only few studies have focused on imaging findings in mucositis. In a clinical study, Vos et al. scanned patients with malignant haematological conditions who had received chemotherapy and found that 86% had 2-[^18^F]FDG uptake in the intestinal wall. Patients who reported abdominal pain had high levels of gastrointestinal 2-[^18^F]FDG uptake. An aim of the study was to evaluate 2-[^18^F]FDG PET/CT as a tool to direct treatment of infection in neutropenic patients and the authors argued that gastrointestinal 2-[^18^F]FDG uptake was a false positive finding, representing mucositis (39). Nijkamp et al. investigated mucositis of the oesophagus following radiation therapy and found a correlation between eosophageal SUV and the grade of oesophagitis (40). Taken together with our study, these results suggest that 2-[^18^F]FDG PET/CT can be used to objectively evaluate the presence of gastrointestinal mucositis, seemingly applicable in a clinical setting. To our knowledge, no other studies have used PET to monitor the time course of mucositis.

The PET scan is non-invasive and enables *in vivo* evaluation of the entire gastrointestinal tract, thus allowing localisation of the inflamed areas. Experimentally, small animal endoscopy is possible but is restricted to the colon and may be challenging to perform in inflamed and friable tissue. 2-[^18^F]FDG-PET/CT overcomes these obstacles and is applicable across species. Even in this murine model with discrete signs of mucositis, the abdominal SUV_BW_ was clearly elevated. Clinically, PET combined with CT or MRI is already available in many tertiary healthcare services, and studies investigating the utility of PET in various infectious and inflammatory conditions have reported both high specificity and sensitivity (41).

The small intestine is not fixed in position in the abdominal cavity. It may be located differently from mouse to mouse and from scan to scan, and quantification of the 2-[^18^F]FDG uptake in the small intestine is not standardised. We found an unequal distribution of ingested contrast throughout the small intestine, even though the mice had received CT contrast in their drinking water two days before each scan. This led us to analyse the abdominal SUV_BW_ value over the entire abdomen, not delineating the small intestine, by placing a spherical VOI over the entire abdomen. We subsequently manually subtracted physiologically high 2-[^18^F]FDG-uptake regions such as kidneys and bladder using the PET images. Skeletal structures were subtracted from the VOI using the CT images. With this approach, we could not identify the location of inflammation in the mobile small intestinal segments, but we did demonstrate a significant difference between the doxorubicin-treated group and the control group. Others have applied different approaches to determine the 2-[^18^F]FDG uptake in the small intestine on PET scans. One approach to detect small ulcerations was to define several signal-intense regions of 1 mm in diameter in the small intestine and derive an abdominal SUV from 50-60 regions of interest for each animal treated with NSAID (19, 42). Another bidirectional approach used both oral gavage and rectal administration of iodine-containing contrast agents. This enabled the use of an isocontour function to automatically generate a region of interest on CT images of the intestine and skeleton, after which skeletal structures were manually removed (43). Our approach was not affected by intestinal movement but was sensitive to changes in the subject size, as the SUV_BW_ is calculated as a mean of values in a volume and thus depends on the volume size. As shown in **Supplementary Figure S5**, the mean volumes of interest tended to be larger in the doxorubicin-treated group on days 1, 6, and 10, but there was no difference on day 3, where the most pronounced difference in abdominal SUV_BW_ was seen. On day 10, there was a broad volume difference, but no difference in SUV_BW_ and the difference in volume led to an underestimation of the actual difference in abdominal SUV_BW_ between the two groups on day 10. Adjusting for the VOI size did not significantly change our findings on days 1-6, but indicated that abdominal uptake in the doxorubicin gorup was still above baseline levels on day 10.

A limitation of our study was a significant accumulation of 2-[^18^F]FDG in the bladder that caused a spillover into the rest of the abdomen, hampering the identification of the PET signal from the intestinal wall. The spillover could possibly have been curtailed by measures of reducing the bladder content, such as manual stimulation of voiding, catheterization, or furosemide-type diuretics (43). Another limitation of our study is that we used a parallel group to obtain tissue for histopathological analysis, and hence were not able to directly correlate the findings on histopathology to the obtained uptake values.

PET/CT or PET combined with MRI are well-established modalities in the clinical setting, which provide a basis for translating our results into clinical practice. Several other mouse models for different chemotherapeutic regimens could be investigated using 2-[^18^F]FDG-PET/CT. We used only a single doxorubicin injection in the present study, but fractionated dose regimens could also be longitudinally examined. PET/CT imaging need more validation in mucositis research, but may represent an objective, quantitative outcome measure. MRI is available for small animal imaging and provides superior soft tissue discrimination, thus allowing even better identification and delineation of the small intestine and possibly enabling more precise anatomical localisation of the inflammation.

## 5 Conclusion

Gastrointestinal mucositis is a highly dynamic and tissue-specific process in which many processes occur simultaneously, representing different stages in mucositis development. This results in synergistic interaction and dysregulation of mucosal homeostasis and repair (44, 45). In a study of mice scanned sequentially with 2-[^18^F]FDG-PET/CT, we found that abdominal SUV_BW_ objectively and semi-quantitatively visualised the development and resolution of doxorubicin-induced mucositis, even in this mild mucositis model. Thus, we obtained valuable information on the time-dependent process of mucositis development and resolution after chemotherapy. This approach may prove valuable for evaluating disease development and severity in future interventional studies or genetic models of chemotherapy-induced gastrointestinal mucositis.

## Data availability statement

The datasets presented in this study can be found in online repositories. The names of the repository/repositories and accession number(s) can be found below: 1 https://doi.org/10.6084/m9.figshare.19411367.v1.

## Ethics statement

The animal study was reviewed and approved by The Danish Animal Experiments Inspectorate, license number: 2017-15-0201-01385.

## Author contributions

SD designed the study, planned the experiments, analysed the data, and drafted the manuscript. SS performed experimental procedures. CB performed experimental procedures and drafted part of the manuscript. LC performed data analysis. MR developed the model, supplied materials and drafted the manuscript. MP performed data analysis. SH designed the study and drafted the manuscript. JM designed the study, supervised and aided with the experiments and data interpretation, and drafted the manuscript. All authors contributed to the article and approved the submitted version.

## References

[1] Al-Dasooqi N Sonis ST Bowen JM Bateman E Blijlevens N Gibson RJ. Emerging evidence on the pathobiology of mucositis. Supportive Care Cancer (2013) 21(7):2075–83. doi: 10.1007/s00520-013-1810-y 23604521

[2] Kuiken NS Rings EH Tissing WJ. Risk analysis, diagnosis and management of gastrointestinal mucositis in pediatric cancer patients. Crit Rev Oncol/Hematol (2014) 24(3):1357–64. doi: 10.1016/j.critrevonc.2014.12.009 25560731

[3] Sonis ST Elting LS Keefe D Peterson DE Schubert M Hauer-Jensen M. Perspectives on cancer therapy-induced mucosal injury: Pathogenesis, measurement, epidemiology, and consequences for patients. Cancer (2004) 100(9 Suppl):1995–2025. doi: 10.1002/cncr.20162 15108222

[4] Villa A Sonis ST. Mucositis: Pathobiology and management. Curr Opin Oncol (2015) 27(3):159–64. doi: 10.1097/CCO.0000000000000180 25774860

[5] Jones JA Avritscher EB Cooksley CD Michelet M Bekele BN Elting LS. Epidemiology of treatment-associated mucosal injury after treatment with newer regimens for lymphoma, breast, lung, or colorectal cancer. Support Care Cancer (2006) 14(6):505–15. doi: 10.1007/s00520-006-0055-4 16601950

[6] Keefe DM. Intestinal mucositis: Mechanisms and management. Curr Opin Oncol (2007) 19(4):323–7. doi: 10.1097/CCO.0b013e3281214412 17545794

[7] Logan RM Stringer AM Bowen JM Gibson RJ Sonis ST Keefe DM. Is the pathobiology of chemotherapy-induced alimentary tract mucositis influenced by the type of mucotoxic drug administered? Cancer Chemother Pharmacol (2009) 63(2):239–51. doi: 10.1007/s00280-008-0732-8 18351341

[8] Wardill HR Sonis ST Blijlevens NMA Van Sebille YZA Ciorba MA Loeffen EAH. Prediction of mucositis risk secondary to cancer therapy: A systematic review of current evidence and call to action. Supportive Care Cancer (2020) 28(11):5059–73. doi: 10.1007/s00520-020-05579-7 32592033

[9] da Silva Ferreira AR Wardill HR Tissing WJE Harmsen HJM. Pitfalls and novel experimental approaches to optimize microbial interventions for chemotherapy-induced gastrointestinal mucositis. Curr Opin Support Palliat Care (2020) 14(2):127–34. doi: 10.1097/SPC.0000000000000497 PMC7259380 32324645

[10] Peterson DE Boers-Doets CB Bensadoun RJ Herrstedt J Committee EG. Management of oral and gastrointestinal mucosal injury: Esmo clinical practice guidelines for diagnosis, treatment, and follow-up. Ann Oncol (2015) 26 Suppl 5:v139–51. doi: 10.1093/annonc/mdv202 26142468

[11] Sonis ST. The pathobiology of mucositis. Nat Rev Cancer (2004) 4(4):277–84. doi: 10.1038/nrc1318 15057287

[12] Bowen J Al-Dasooqi N Bossi P Wardill H Van Sebille Y Al-Azri A. The pathogenesis of mucositis: Updated perspectives and emerging targets. Support Care Cancer (2019) 27(10):4023–33. doi: 10.1007/s00520-019-04893-z 31286231

[13] Menezes-Garcia Z do Nascimento Arifa R Fagundes C Souza D. Mechanisms underlying chemotherapy-associated mucositis: The role of inflammatory mediators and potential therapeutic targets. EMJ Gastroenterol (2018) 7):82–91. doi: 10.33590/emjgastroenterol/10310983

[14] Sangild PT Shen RL Pontoppidan P Rathe M. Animal models of chemotherapy-induced mucositis: Translational relevance and challenges. Am J Physiol Gastrointest Liver Physiol (2018) 314(2):G231–G46. doi: 10.1152/ajpgi.00204.2017 29074485

[15] Wardill HR Tissing WJE Kissow H Stringer AM. Animal models of mucositis: Critical tools for advancing pathobiological understanding and identifying therapeutic targets. Curr Opin Supportive Palliative Care (2019) 13(2):119–33. doi: 10.1097/Spc.0000000000000421 30925531

[16] de Koning BA Lindenbergh-Kortleve DJ Pieters R Buller HA Renes IB Einerhand AW. Alterations in epithelial and mesenchymal intestinal gene expression during doxorubicin-induced mucositis in mice. Dig Dis Sci (2007) 52(8):1814–25. doi: 10.1007/s10620-006-9174-5 PMC1914222 17415656

[17] Bech AS Nexoe AB Dubik M Moeller JB Soerensen GL Holmskov U. Peptidoglycan recognition peptide 2 aggravates weight loss in a murine model of chemotherapy-induced gastrointestinal toxicity. Front Oncol (2021) 11:635005. doi: 10.3389/fonc.2021.635005 33833993 PMC8021894

[18] Tang TT Rendon DA Zawaski JA Afshar SF Kaffes CK Sabek OM. Imaging radiation-induced gastrointestinal, bone marrow injury and recovery kinetics using 18f-fdg pet. PloS One (2017) 12(1):e0169082. doi: 10.1371/journal.pone.0169082 28052129 PMC5214459

[19] Yamato M Kataoka Y Mizuma H Wada Y Watanabe Y. Pet and macro- and microautoradiographic studies combined with immunohistochemistry for monitoring rat intestinal ulceration and healing processes. J Nucl Med (2009) 50(2):266–73. doi: 10.2967/jnumed.108.057943 19164236

[20] Cusso L Reigadas E Munoz P Desco M Bouza E. Evaluation of clostridium difficile infection with Pet/Ct imaging in a mouse model. Mol Imaging Biol (2020) 22(3):587–92. doi: 10.1007/s11307-019-01408-4 31317298

[21] Smith AJ Clutton RE Lilley E Hansen KEA Brattelid T. Prepare: Guidelines for planning animal research and testing. Lab Anim (2018) 52(2):135–41. doi: 10.1177/0023677217724823 PMC5862319 28771074

[22] Percie du Sert N Hurst V Ahluwalia A Alam S Avey MT Baker M. The arrive guidelines 2.0: Updated guidelines for reporting animal research. BMC Vet Res (2020) 16(1):242. doi: 10.1186/s12917-020-02451-y 32660541 PMC7359286

[23] Steward JP Ornellas EP Beernink KD Northway WH. Errors in the technique of intraperitoneal injection of mice. Appl Microbiol (1968) 16(9):1418–9. doi: 10.1128/am.16.9.1418-1419.1968 PMC547667 5676408

[24] Arioli V Rossi E. Errors related to different techniques of intraperitoneal injection in mice. Appl Microbiol (1970) 19(4):704–5. doi: 10.1128/am.19.4.704-705.1970 PMC376768 5418953

[25] Chiu CJ McArdle AH Brown R Scott HJ Gurd FN. Intestinal mucosal lesion in low-flow states. i. a morphological, hemodynamic, and metabolic reappraisal. Arch Surg (1970) 101(4):478–83. doi: 10.1001/archsurg.1970.01340280030009 5457245

[26] Stringer AM Al-Dasooqi N Bowen JM Tan TH Radzuan M Logan RM. Biomarkers of chemotherapy-induced diarrhoea: A clinical study of intestinal microbiome alterations, inflammation and circulating matrix metalloproteinases. Support Care Cancer (2013) 21(7):1843–52. doi: 10.1007/s00520-013-1741-7 23397098

[27] Logan RM Gibson RJ Bowen JM Stringer AM Sonis ST Keefe DM. Characterisation of mucosal changes in the alimentary tract following administration of irinotecan: Implications for the pathobiology of mucositis. Cancer Chemother Pharmacol (2008) 62(1):33–41. doi: 10.1007/s00280-007-0570-0 17703303

[28] Nexoe AB Pedersen AA von Huth S Sorensen GL Holmskov U Jiang PP. No effect of deleted in malignant brain tumors 1 deficiency on chemotherapy induced murine intestinal mucositis. Sci Rep (2021) 11(1):14687. doi: 10.1038/s41598-021-94076-w 34282203 PMC8289998

[29] Andersen MCE Johansen MW Nissen T Nexoe AB Madsen GI Sorensen GL. Fibcd1 ameliorates weight loss in chemotherapy-induced murine mucositis. Support Care Cancer (2021) 29(5):2415–21. doi: 10.1007/s00520-020-05762-w 32918133

[30] Shen RL Pontoppidan PE Rathe M Jiang P Hansen CF Buddington RK. Milk diets influence doxorubicin-induced intestinal toxicity in piglets. Am J Physiol Gastrointest Liver Physiol (2016) 311(2):G324–33. doi: 10.1152/ajpgi.00373.2015 27445347

[31] Chen C Zhang Q Yu W Chang B Le AD. Oral mucositis: An update on innate immunity and new interventional targets. J Dent Res (2020) 99(10):1122–30. doi: 10.1177/0022034520925421 PMC7443999 32479139

[32] Iking J Staniszewska M Kessler L Klose JM Luckerath K Fendler WP. Imaging inflammation with positron emission tomography. Biomedicines (2021) 9(2):212. doi: 10.3390/biomedicines9020212 33669804 PMC7922638

[33] Eichele DD Kharbanda KK. Dextran sodium sulfate colitis murine model: An indispensable tool for advancing our understanding of inflammatory bowel diseases pathogenesis. World J Gastroenterol (2017) 23(33):6016–29. doi: 10.3748/wjg.v23.i33.6016 PMC5597494 28970718

[34] Hindryckx P Staelens S Devisscher L Deleye S De Vos F Delrue L. Longitudinal quantification of inflammation in the murine dextran sodium sulfate-induced colitis model using Mupet/Ct. Inflammation Bowel Dis (2011) 17(10):2058–64. doi: 10.1002/ibd.21578 21910167

[35] Bettenworth D Reuter S Hermann S Weckesser M Kerstiens L Stratis A. Translational 18f-fdg Pet/Ct imaging to monitor lesion activity in intestinal inflammation. J Nucl Med (2013) 54(5):748–55. doi: 10.2967/jnumed.112.112623 23516311

[36] Okayasu I Hatakeyama S Yamada M Ohkusa T Inagaki Y Nakaya R. A novel method in the induction of reliable experimental acute and chronic ulcerative colitis in mice. Gastroenterology (1990) 98(3):694–702. doi: 10.1016/0016-5085(90)90290-h 1688816

[37] Seoane-Viano I Gomez-Lado N Lazare-Iglesias H Barreiro-de Acosta M Silva-Rodriguez J Luzardo-Alvarez A. Longitudinal Pet/Ct evaluation of tnbs-induced inflammatory bowel disease rat model. Int J Pharm (2018) 549(1-2):335–42. doi: 10.1016/j.ijpharm.2018.08.005 30081226

[38] Adams MC Turkington TG Wilson JM Wong TZ. A systematic review of the factors affecting accuracy of suv measurements. AJR Am J Roentgenol (2010) 195(2):310–20. doi: 10.2214/AJR.10.4923 20651185

[39] Vos FJ Donnelly JP Oyen WJ Kullberg BJ Bleeker-Rovers CP Blijlevens NM. 18f-fdg Pet/Ct for diagnosing infectious complications in patients with severe neutropenia after intensive chemotherapy for haematological malignancy or stem cell transplantation. Eur J Nucl Med Mol Imaging (2012) 39(1):120–8. doi: 10.1007/s00259-011-1939-1 PMC3227801 21947022

[40] Nijkamp J Rossi M Lebesque J Belderbos J van den Heuvel M Kwint M. Relating acute esophagitis to radiotherapy dose using fdg-pet in concurrent chemo-radiotherapy for locally advanced non-small cell lung cancer. Radiother Oncol (2013) 106(1):118–23. doi: 10.1016/j.radonc.2012.09.024 23219463

[41] Hess S Hansson SH Pedersen KT Basu S Hoilund-Carlsen PF. Fdg-Pet/Ct in infectious and inflammatory diseases. PET Clin (2014) 9(4):497–519. doi: 10.1016/j.cpet.2014.07.002 26050949

[42] Zhuang H Alavi A. 18-fluorodeoxyglucose positron emission tomographic imaging in the detection and monitoring of infection and inflammation. Semin Nucl Med (2002) 32(1):47–59. doi: 10.1053/snuc.2002.29278 11839069

[43] Brewer S McPherson M Fujiwara D Turovskaya O Ziring D Chen L. Molecular imaging of murine intestinal inflammation with 2-Deoxy-2-[18f]Fluoro-D-Glucose and positron emission tomography. Gastroenterology (2008) 135(3):744–55. doi: 10.1053/j.gastro.2008.06.040 PMC3752978 18639553

[44] Bowen JM Gibson RJ Tsykin A Stringer AM Logan RM Keefe DM. Gene expression analysis of multiple gastrointestinal regions reveals activation of common cell regulatory pathways following cytotoxic chemotherapy. Int J Cancer (2007) 121(8):1847–56. doi: 10.1002/ijc.22895 17594691

[45] Rathe M Thomassen M Shen RL Pontoppidan PE Husby S Muller K. Chemotherapy modulates intestinal immune gene expression including surfactant protein-d and deleted in malignant brain tumors 1 in piglets. Chemotherapy (2016) 61(4):204–16. doi: 10.1159/000442938 26886263

