# Supplementary material for: PET/CT imaging detects intestinal inflammation in a mouse model of doxorubicin-induced mucositis
Source: Front Oncol. 2022 Dec 15;12:1061804. doi: 10.3389/fonc.2022.1061804 (PMC9798215; doi:10.3389/fonc.2022.1061804)
Supplement: Supplementary file 1 [file DataSheet_1.docx]

PET/CT imaging detects intestinal inflammation in a mouse model of doxorubicin-induced mucositis

**Supplementary materials**

**Supplementary Fig. S1**

Representative example of volume of interest delineated in a mouse scanned at baseline.


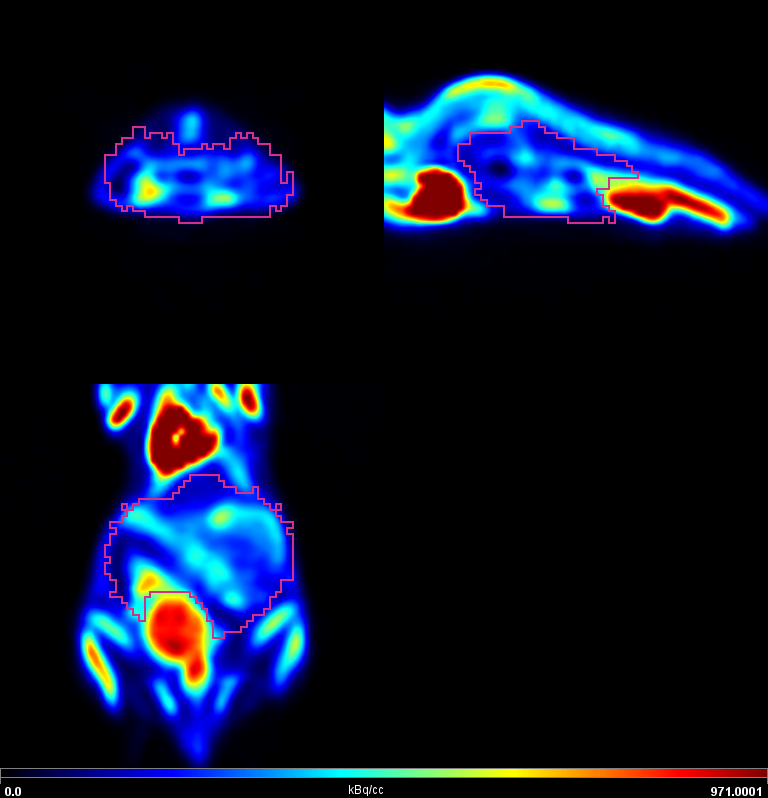


**Supplementary Fig. S2**

Crypt depth of the colon. There was no difference between the control and the doxorubicin-treated group. For controls: Day 1, n = 4; day 3, n = 4: day 6, n = 4; day 10, n = 7. For doxorubicin: Day 1, n = 7; day 3, n = 8; day 6, n = 6; and day 10, n = 16. Data presented as means ± sd with baseline levels indicated by the dashed line.

**Supplementary Fig. S3**

Length of the small intestine and the colon. There were no differences between the two groups on any day using two-way ANOVA with Holm-Šídák’s multiple comparisons test: p > 0.05 on all days. For controls: Day 1, n = 4; day 3, n = 4: day 6, n = 3 (one tissue specimen too fragmented to measure); day 10, n = 7. For doxorubicin: Day 1, n = 7; day 3, n = 8; day 6, n = 6; and day 10, n = 16. Data presented as means ± sd with baseline levels indicated by the dashed line.

**Supplementary Fig. S4**

Weight of the stomach and the spleen. There were no differences between the control and the doxorubicin groups on any day using two-way ANOVA with Holm-Šídák’s multiple comparisons test: p > 0.05 on all days. For controls: Day 1, n = 4; day 3, n = 4: day 6, n = 4; day 10, n = 7. For doxorubicin: Day 1, n = 7; day 3, n = 8; day 6, n = 6; and day 10, n = 16. Data presented as means ±SD with baseline levels indicated by the dashed line.

**Supplementary Fig. S5**

Comparison of volume of interest (VOI) size between the doxorubicin-treated group and the control group. Mean and standard deviation indicated by horizontal lines.

**Supplementary Fig. S6**

2-[^18^F]FDG uptake values times the size of the volume of interest as a measure of the total uptake.

**Supplementary Fig. S7**

Blood glucose levels before each scan. Mean and standard deviation are indicated by horizontal lines.
